# Smartphone-Based Escalator Recognition for the Visually Impaired

**DOI:** 10.3390/s17051057

**Published:** 2017-05-06

**Authors:** Daiki Nakamura, Hotaka Takizawa, Mayumi Aoyagi, Nobuo Ezaki, Shinji Mizuno

**Affiliations:** 1 Department of Computer Science, University of Tsukuba, 1-1-1 Tennodai, Tsukuba 305-8573, Japan; nakamura.at@pr.cs.tsukuba.ac.jp; 2 Aichi University of Education, 1 Hirosawa, Igaya, Kariya 448-8542, Japan; maoyagi@auecc.aichi-edu.ac.jp; 3 Toba National College of Maritime Technology, 1-1 Ikegami, Toba 517-8501, Japan; ezaki@toba-cmt.ac.jp; 4 Aichi Institute of Technology, 1247 Yachigusa, Yakusa, Toyota 470-0392, Japan; s_mizuno@aitech.ac.jp

**Keywords:** Assistive system, Visually impaired user, Escalator, Optical flow, Smartphone camera

## Abstract

It is difficult for visually impaired individuals to recognize escalators in everyday environments. If the individuals ride on escalators in the wrong direction, they will stumble on the steps. This paper proposes a novel method to assist visually impaired individuals in finding available escalators by the use of smartphone cameras. Escalators are recognized by analyzing optical flows in video frames captured by the cameras, and auditory feedback is provided to the individuals. The proposed method was implemented on an Android smartphone and applied to actual escalator scenes. The experimental results demonstrate that the proposed method is promising for helping visually impaired individuals use escalators.

## 1. Introduction

In 2014, the World Health Organization reported that the number of visually impaired individuals was estimated to be approximately 285 million worldwide [1]. Many of them use white canes to detect obstacles ahead of them, but the detection ranges are short. Guide dogs are also used for navigation, but they need long training periods and large budgets. Therefore, it is necessary to build assistive systems [2] to help the visually impaired.

Several research groups have proposed cane-type systems [3,4,5,6,7,8,9,10,11,12], belt-type systems [13,14,15,16], helmet-type systems [17,18,19,20], wearable systems [21,22,23], glasses-type systems [24,25], and robot systems [26,27,28,29] to detect obstacles and recognize objects in environments. These assistive systems were handmade; therefore, it is difficult for visually impaired individuals to obtain them.

Other research groups have proposed assistive systems based on general smartphones. Obstacle detection systems were proposed in [14,26,30]. Dumitras et al. [31] proposed a mobile text-recognition system to allow the visually impaired to access text information. Tekin et al. [32] developed a system to detect and read LED/LCD digit characters of a certain font. Zhang et al. [33] proposed a mobile recognition system of braille characters [34] on public telephones or guide plates. Sara et al. [35] built a color recognition system for clothing coordination based on HSL color space processing. Matusiak et al. [36] proposed a recognition system of food or medicine packages based on Scale-Invariant Feature Transform (SIFT) [37] or Features from accelerated segment test (FAST) [38]. Ivanchenko et al. proposed a mobile phone system to allow the visually impaired to know the positions of crosswalks [39]. They also proposed a walk light detection system to let visually impaired individuals know when it is time to cross [40]. These systems can recognize static objects around visually impaired individuals. However, in real environments, there are many dynamic objects such as people and cars.

Tapu et al. [41,42,43] proposed categorization methods of dynamic objects such as cars, bicycles, and pedestrians, as well as static obstructions based on computer vision techniques. The methods were implemented on portable systems composed of smartphones and several devices mounted on chest harnesses. These systems can notify visually impaired individuals about dynamic objects, but cannot help the individuals use the objects. The systems can only warn the individuals not to collide with the objects. In daily life, however, even visually impaired individuals often need to use dynamic objects, such as moving walkways and rotating doors.

In this paper, we focus on escalators. In general, visually impaired individuals estimate the positions of escalators based on motor sounds and then walk to the estimated positions. Subsequently, they grope for the belts of the escalators, and confirm their movement directions. If the directions are suitable, they can ride on the escalators. In actual escalator scenes, however, it is difficult to find the escalator belts; therefore, the individuals often fail to determine the movement directions. If the escalators move in the wrong directions, it can be dangerous for the visually impaired.

This paper proposes an escalator recognition method for visually impaired individuals. This method can detect the positions of escalators and determine their movement directions from videos obtained with a smartphone camera. The method can also provide auditory feedback to let the individuals know the recognition results. The proposed method is implemented on an Android smartphone.

Section 2 describes the outline of the proposed method, Section 3 shows experimental results from actual escalator scenes, Section 4 discusses the proposed method, and Section 5 concludes the paper.

## 2. Outline of the Proposed Method

**Figure 1:**
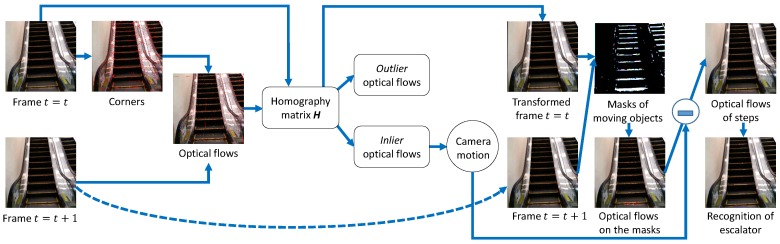
Outline of the proposed method.

**Figure 2:**
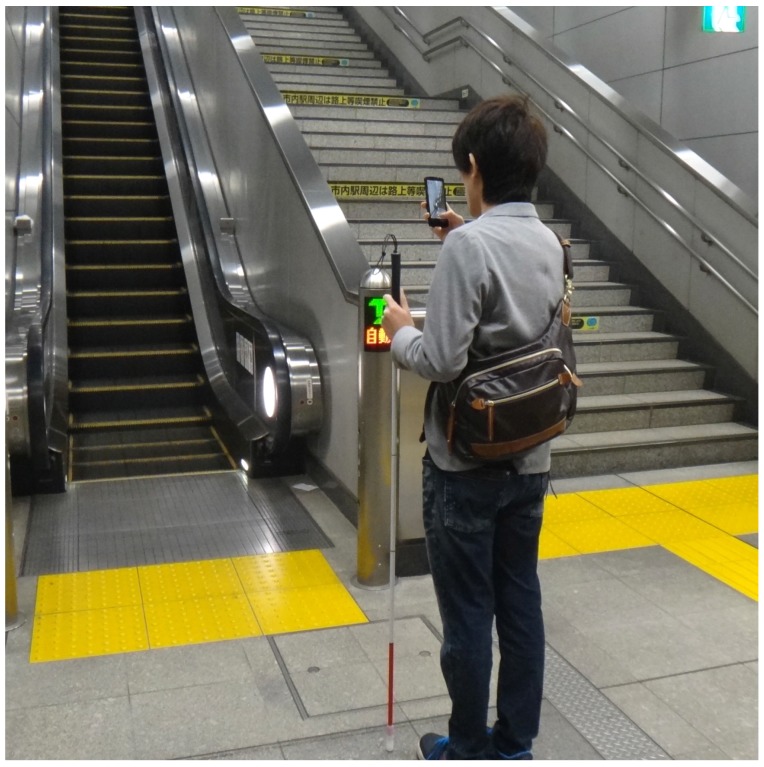
A user takes a video with a smartphone camera.

Figure 1 shows the outline of the proposed method. First, when a visually impaired user predicts that he or she is in front of an escalator, the user sets his or her smartphone vertically and takes a video of the scene with the camera, as shown in Figure 2. The user can pan the camera to search for an escalator, if necessary. The video is divided into frames, from which corner points are detected. Optical flows are computed at the corners in two successive frames. A homography matrix **H** is estimated by applying the random sample consensus (RANSAC) algorithm [44] to the optical flows, which are classified into two categories: *inliers* and *outliers*. The inlier optical flows come from the camera motion, because it affects the entire image. The camera motion is obtained by averaging the inlier optical flows. The frame at t=t is transformed into the frame at t=t+1 by using an image registration technique based on the Homography matrix. A difference image is made from the transformed frame at t=t and the frame at t=t+1. In the difference image, moving objects appear as regions with high intensities. These regions are extracted as masks by a binarization operator followed by morphological operations [45] for shape smoothing. The camera motion is subtracted from the optical flows on the masks in the frame at t=t+1. The final optical flows represent the direction of the moving objects (i.e., steps). Depending on the number and direction of the optical flows, the system recognizes the escalator and informs the user.

The method is described in detail in the following sections.

### 2.1. Corner Detection

Escalator steps have concave–convex surfaces for skidproof purposes, and are highlighted with an accent color such as yellow. Therefore, they are often observed as a set of corner points with strong contrast in video frames. Such corner points are detected as described below.

Let *I(x,y,t)* denote the intensity of a pixel p(x,y) in a frame at a certain time *t*, and $\lambda_{p}$ denote the minimal eigenvalue of the following matrix [46]:

(1) $$M=\left[\begin{array}{cc} \sum_{S_{p}}^{}{\left(\frac{dI}{dx}\right)}^{2} & \sum_{S_{p}}^{}\left(\frac{dI}{dx}\frac{dI}{dy}\right)\\ \sum_{S_{p}}^{}\left(\frac{dI}{dx}\frac{dI}{dy}\right) & \sum_{S_{p}}^{}{\left(\frac{dI}{dy}\right)}^{2} \end{array}\right],$$

where $S_{p}$ represents a small region with its center at pixel *p* in the frame. The minimal eigenvalues are calculated at all the pixels, and if a minimal eigenvalue $\lambda_{p}$ is the maximum in $S_{p}$, it is eliminated. Among the remaining eigenvalues, the maximum eigenvalue $\lambda_{max}$ is determined, and eigenvalues less than $Q_{\lambda }$% of $\lambda_{max}$ are also eliminated. The remaining eigenvalues are denoted by $\lambda_{\left(1\right)}$, $\lambda_{\left(2\right)}$, ⋯ ($\lambda_{\left(1\right)}\geq \lambda_{\left(2\right)}\geq \cdots$), where $\lambda_{\left(1\right)}$ is equivalent to $\lambda_{max}$. The pixel at $\lambda_{max}$ is extracted as the first corner point. If the distance between $\lambda_{\left(i\right)}$ and $\lambda_{\left(j\right)}$ (i>j) is larger than a predefined threshold $d_{cd}$, the pixel at $\lambda_{\left(i\right)}$ is also extracted as a corner point. In this way, $N_{fn}$ corner points are extracted. The corner points can be used as clues for the recognition of the escalator steps.

### 2.2. Optical Flow Computation

In order to recognize the movement direction of the escalator steps, we used the gradient-based optical flow detection method, where the intensities of corresponding pixels are assumed to remain unchanged in two successive frames. The assumption is represented by

(2) I(x,y,t)=I(x+δx,y+δy,t+δt),

where (δx,δy) is the displacement of a pixel p(x,y) during an interval time δt. By applying the Taylor-expansion to the right term of Equation (2), we can obtain

(3) $$I\left(x+\delta x,y+\delta y,t+\delta t\right)=I\left(x,y,t\right)+\frac{\partial I}{\partial x}\delta x+\frac{\partial I}{\partial y}\delta y+\frac{\partial I}{\partial t}\delta t+O_{I},$$

where $O_{I}$ is a high-order term, which can be omitted. From Equations (2) and (3), the following equation is obtained:

(4) $$\frac{\partial I}{\partial x}\frac{\delta x}{\delta t}+\frac{\partial I}{\partial y}\frac{\delta y}{\delta t}+\frac{\partial I}{\partial t}=0.$$

Let $u_{x}$ and $u_{y}$ be the *x* and *y* components of the optical flow at p(x,y), respectively, and $I_{x}$, $I_{y}$, and $I_{t}$ be the derivatives of I(x,y,t) in the corresponding directions. By using $u_{x}$, $u_{y}$, $I_{x}$, $I_{y}$, and $I_{t}$, Equation (4) is converted to

(5) $$I_{x}u_{x}+I_{y}u_{y}+I_{t}=0.$$

Equation (5) is known as the optical flow constraint equation, which has two variables: $u_{x}$ and $u_{y}$. This equation can be solved with the Lucas–Kanade algorithm (LKA) [47], which assumes that optical flows are uniform in local regions. Let us consider a small region whose center is at p(x,y). The region size is set to be $N_{rs}=M_{rs}\times M_{rs}$ pixels. The assumption gives us the following $N_{rs}$ equations:

(6) $$\begin{array}{c} \left\{\begin{array}{c} I_{x}^{\left(1\right)}u_{x}+I_{y}^{\left(1\right)}u_{y}=-I_{t}^{\left(1\right)},\\ I_{x}^{\left(2\right)}u_{x}+I_{y}^{\left(2\right)}u_{y}=-I_{t}^{\left(2\right)},\\ \;\;\vdots \\ I_{x}^{\left(N_{rs}\right)}u_{x}+I_{y}^{\left(N_{rs}\right)}u_{y}=-I_{t}^{\left(N_{rs}\right)}. \end{array}\right. \end{array}$$

These equations are rewritten as follows:

(7) Au=−b,

where

(8) $$A=\left[\begin{array}{cc} I_{x}^{\left(1\right)} & I_{y}^{\left(1\right)}\\ \vdots & \vdots \\ I_{x}^{\left(N_{rs}\right)} & I_{y}^{\left(N_{rs}\right)} \end{array}\right],\;u=\left[\begin{array}{c} u_{x}\\ u_{y} \end{array}\right],\;b=\left[\begin{array}{c} I_{t}^{\left(1\right)}\\ \vdots \\ I_{t}^{\left(N_{rs}\right)} \end{array}\right].$$

The optical flow u can be obtained by applying the least squares method to Equation (7). The LKA is sensitive to noise in the frames; therefore, we used the extended LKA [48] based on the pyramidal multiresolution analysis, where optical flows in a frame at a resolution are computed from those in another frame at a lower resolution.

### 2.3. Homography Transformation for Image Registration

Frames are often deformed due to accidental movement of the user’s hands when taking the video. The deformation is compensated by the homography transformation [49], which is a kind of planar projective transformation. Let ${\left(x_{i},y_{i},w_{i}\right)}^{T}$ and ${\left(x_{i}^{\prime },y_{i}^{\prime },w_{i}^{\prime }\right)}^{T}$ denote the 2D homogeneous coordinates of the start and end points of the *i*-th optical flow, respectively $\left(i=1,2,\cdots ,N_{fn}\right)$. The homography transformation is represented by

(9) $$\left[\begin{array}{c} x_{i}^{\prime }\\ y_{i}^{\prime }\\ w_{i}^{\prime } \end{array}\right]=\left[\begin{array}{ccc} h_{1} & h_{2} & h_{3}\\ h_{4} & h_{5} & h_{6}\\ h_{7} & h_{8} & h_{9} \end{array}\right]\left[\begin{array}{c} x_{i}\\ y_{i}\\ w_{i} \end{array}\right],$$

where

(10) $$H=\left[\begin{array}{ccc} h_{1} & h_{2} & h_{3}\\ h_{4} & h_{5} & h_{6}\\ h_{7} & h_{8} & h_{9} \end{array}\right],$$

is known as the homography matrix and is computed using the direct linear transformation (DLT) algorithm [49].

#### 2.3.1. DLT Algorithm

In the DLT algorithm, the homography transformation is represented by

(11) $$\left[\begin{array}{ccccccccc} x_{1} & y_{1} & 1 & 0 & 0 & 0 & -x_{1}x_{1}^{\prime } & -y_{1}x_{1}^{\prime } & -x_{1}^{\prime }\\ 0 & 0 & 0 & x_{1} & y_{1} & 1 & -x_{1}y_{1}^{\prime } & -y_{1}y_{1}^{\prime } & -y_{1}^{\prime }\\ x_{2} & y_{2} & 1 & 0 & 0 & 0 & -x_{2}x_{2}^{\prime } & -y_{2}x_{2}^{\prime } & -x_{2}^{\prime }\\ 0 & 0 & 0 & x_{2} & y_{2} & 1 & -x_{2}y_{2}^{\prime } & -y_{2}y_{2}^{\prime } & -y_{2}^{\prime }\\ & \vdots & & & \vdots & & & \vdots & \end{array}\right]\left[\begin{array}{c} h_{1}\\ h_{2}\\ h_{3}\\ h_{4}\\ h_{5}\\ h_{6}\\ h_{7}\\ h_{8}\\ h_{9} \end{array}\right]=0,$$

and it is rewritten as

(12) Ah=0,

where **A** is a $2N_{fn}\times 9$ matrix. The parameter vector **h** is another expression of the homography matrix, and can be obtained by the singular value decomposition (SVD) [50], which converts the matrix as follows:

(13) $$A={\mathrm{UDV}}^{T},$$

where **U** is a $2N_{fn}\times 9$ orthogonal matrix, **D** is a 9×9 diagonal matrix, and **V** is a 9×9 orthogonal matrix. Each diagonal element $d_{i}\left(d_{1}\geq d_{2}\geq \cdots \geq d_{i}\geq \cdots \geq d_{9}\geq 0\right)$ of **D** is a singular value of **A**, and also the square root of the eigenvalue of $A^{T}A$. The *i*-th row vector of **V** corresponds to $d_{i}$, and the 9-th row vector is the least squares solution of the homography parameter **h**. Finally, the parameters in **H** are normalized so that $h_{9}=1$.

#### 2.3.2. Estimation of Homography Matrix Using RANSAC

The RANSAC algorithm can estimate reasonable fitting parameters, even from data including outliers. The algorithm is performed as follows.

1. Select four optical flows randomly.
2. Calculate the homography matrix **H** by applying the DLT algorithm to the four optical flows.
3. Count the number of optical flows with back projection errors less than a certain value ε as follows:
  (14) $${\left(x^{\prime }-\frac{h_{1}x+h_{2}y+h_{3}}{h_{7}x+h_{8}y+h_{9}}\right)}^{2}+{\left(y^{\prime }-\frac{h_{4}x+h_{5}y+h_{6}}{h_{7}x+h_{8}y+h_{9}}\right)}^{2}<\varepsilon .$$
  The optical flows which satisfy Equation (14) are determined to be inliers, and the others are determined to be outliers.
4. Iterate the above steps from 1 to 3 for a certain time.
5. Determine the pre-optimal homography matrix that produces the most inliers.
6. Calculate the optimal homography matrix from the inliers of the pre-optimal homography matrix.

Most inliers originate from the camera motion, whereas most outliers originate from moving objects or false optical flows.

### 2.4. Extraction of Optical Flows on Moving Steps

**Figure 3:**
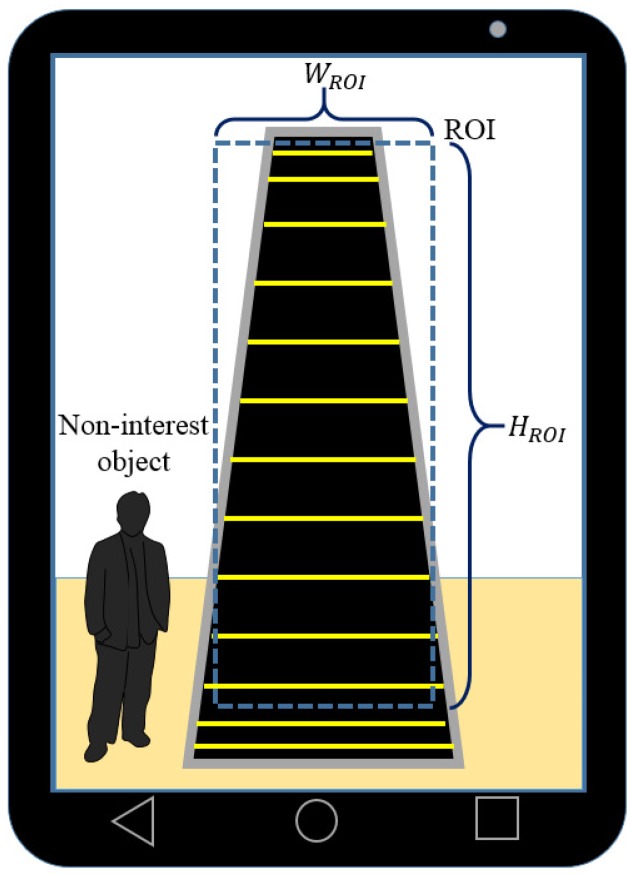
Region of interest (ROI) for escalator recognition.

The frame at t=t is transformed on the basis of the optimal homography matrix. The image subtraction is performed between the transformed frame and the frame at t=t+1. The moving steps appear as regions with high intensities in the subtraction image. These regions are extracted by a binarization operation followed by the closing and opening operations of the mathematical morphology for shape smoothing. The extracted regions are used as masks to select the optical flows on moving steps. A rectangular region of interest (ROI) of $H_{ROI}\times W_{ROI}$ pixels is set on the middle area of the frames to exclude unnecessary optical flows caused by non-interest objects such as people, as shown in Figure 3. The optical flows on the masks in the ROI are extracted to recognize the escalator.

### 2.5. Recognition of an Escalator

Escalators are categorized into the following four classes according to their movement directions:

1. Escalators going to upper floors (denoted by $E_{TU}$)
2. Escalators going to lower floors ($E_{TL}$)
3. Escalators coming from upper floors ($E_{FU}$)
4. Escalators coming from lower floors ($E_{FL}$)

**Figure 4:**
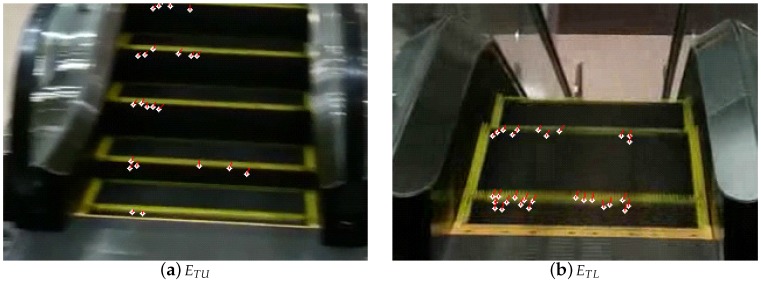
The final optical flows on $E_{TU}$ and $E_{TL}$ escalators.

The escalator classes are determined from the inliers optical flows. First, the camera motion vector is obtained by averaging the inliers optical flows. All the optical flows are subtracted by the camera motion vector. From the subtracted flows, the false optical flows with lengths more than $L_{of}^{u}$ or less than $L_{of}^{l}$ are eliminated. The final optical flows represent the movement direction of the steps of the escalator. If the movement direction is upward in the frame, the escalator is determined to be $E_{TU}$ or $E_{TL}$. Otherwise, it is determined to be $E_{FU}$ or $E_{FL}$. Note that $E_{TL}$ and $E_{FL}$ escalators produce upward and downward optical flows in video frames, respectively. Figure 4 shows the final optical flows on $E_{TU}$ and $E_{TL}$ escalators. White circles and red lines represent the corners and the optical flows, respectively. The $E_{TL}$ and $E_{TU}$ escalators produce upward optical flows.

Next, further classification is performed on the basis of the numbers of steps observed in the frames. The step numbers are obtained from the numbers of groups where the vertical distance between two corner points is closer than $D_{cp}$. If the step numbers are larger than $N_{step}$, the escalator is determined to be $E_{TU}$ or $E_{FU}$. Otherwise, it is determined to be $E_{TL}$ or $E_{FL}$.

### 2.6. Notification to a User

Visually impaired users can use $E_{TU}$ and $E_{TL}$ escalators to move to upper and lower floors, respectively, whereas they cannot use $E_{FU}$ and $E_{FL}$ escalators. The system provides navigation sounds with higher and middle frequencies for $E_{TU}$ and $E_{TL}$, respectively. The system also provides warning sounds with a lower frequency for $E_{FU}$ and $E_{FL}$. The users can select navigation and warning sounds from several sound patterns and also adjust the sound frequencies beforehand so that they can distinguish the sounds effectively.

## 3. Experiments

### 3.1. Conditions

We used the Android smartphone Nexus 5 [51] with a Full High Definition touchscreen. In the corner detection process, the size of $S_{p}$ was set to 3×3. $Q_{\lambda }$, $d_{cd}$, and $N_{fn}$ were set to 0.05%, 5 pixels, and 5000, respectively. In the optical flow computation process, $M_{rs}$ was set to 3 pixels. The ROI sizes $H_{ROI}$ and $W_{ROI}$ were set to 1024 and 120, respectively. In the escalator recognition process, $L_{of}^{u}$, $L_{of}^{l}$, $D_{cp}$, and $N_{steps}$ were set to 8, 2, 24, and 6, respectively.

**Figure 5:**
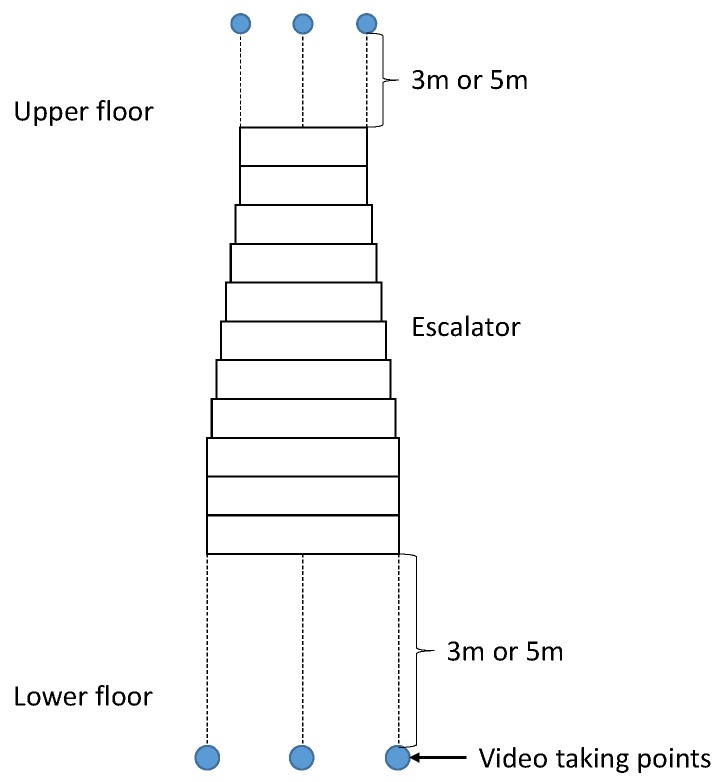
Video taking points near an escalator.

**Figure 6:**
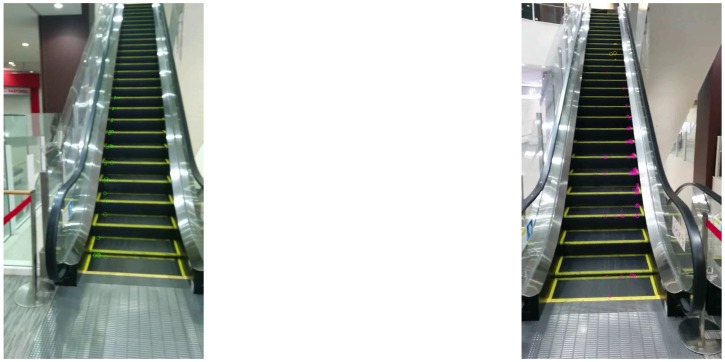
$E_{TU}$ (**left**) and $E_{FU}$ (**right**) escalators observed at 3 m.

**Figure 7:**
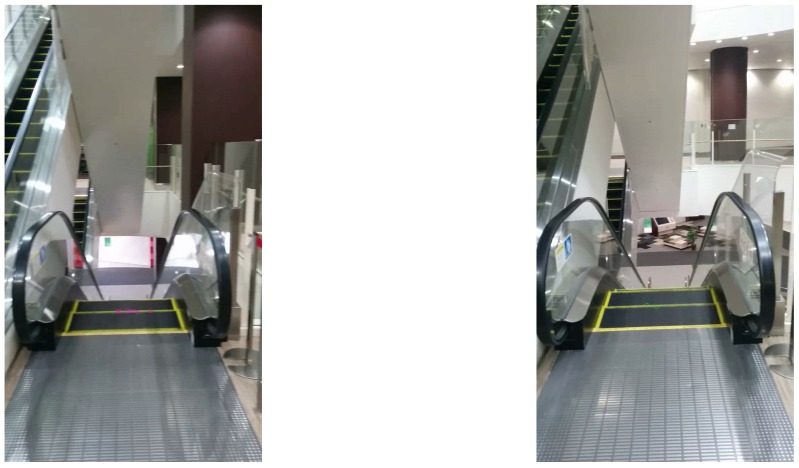
$E_{FL}$ (**left**) and $E_{TL}$ (**right**) escalators observed at 3 m.

The system was evaluated using pre-recorded video frames taken at six points near six escalators as depicted in Figure 5. The smartphone was set at 3 or 5 m from the gates of the escalators and panned horizontally to include the whole of the escalator in the video frames. Figure 6 and Figure 7 are sample escalators. We also used video frames from 24 scenes that did not include any escalators but included several moving objects such as humans, cars, and bikes.

### 3.2. Results

Table 1 and Table 2 list the recognition results of escalators at 3 and 5 m, respectively. The system recognized 97% of $E_{TU}$ and $E_{FU}$ escalators correctly. In contrast, it failed to recognize 18% of $E_{TL}$ and $E_{FL}$ escalators. The $E_{TL}$ and $E_{FL}$ escalators were observed from upper floors, as shown in Figure 7. The system was not able to obtain a sufficient number of corner points and optical flows, which made recognition unstable. On the other hand, the system was able to correctly recognize all the videos of scenes that did not include escalators.

## 4. Discussion

The technical contribution of this paper is to adequately combine the image processing algorithms such as the corner detection, optical flow computation, and image registration. Although they are existing algorithms, the integrated method can do the task (i.e., escalator recognition), which has not been achieved by the previously proposed methods. In Section 3, we designed the experiments considering the variation of the relative distances and directions of users against escalators. The analysis results demonstrated that the proposed method was effective for escalator recognition.

The contribution from a welfare point of view is to be able to help visually impaired individuals use escalators that are representative dynamic objects in daily life. In this paper, we adopted the optical flow analysis to recognize escalators. This analysis method can be applied to other dynamic objects, and would make the lives of the individuals more convenient.

Our preliminary investigation revealed that many visually impaired individuals identified escalators by listening to mechanical sounds, then determined their movement directions by touching the belts. This can be dangerous. By using the proposed system, escalators can be recognized more safely.

The proposed system would not be able to work well in crowded environments such as stations in rush hour times, because other passengers on escalators would produce optical flows that are different from those of the steps. The different optical flows cause misrecognition. The current system cannot deal with this problem; therefore, users should determine the environments by hearing and select whether or not to use the system. In the future, this problem should be solved by eliminating optical flows in human regions extracted by human detection methods such as the histograms of oriented gradients (HOG) technique [52]. In addition, some passengers may not like to have their pictures taken with smartphone cameras. It is not a technical issue, but social understanding is needed.

The proposed method assumes that there are salient features on the steps of escalators. Such features were detected by the corner detection method, and used for optical flow computation. In many countries, escalator steps would have such features to give cautions to passengers, but there are different escalators in the world. It is necessary to improve the system to recognize such escalators correctly.

The proposed method was implemented on a Nexus 5 smartphone, and the processing rate was approximately one frame per second. The processing speed should be increased to make the system more practical.

One of the authors is blind. The author mentioned that it was easy to take the pictures of escalators because the author can know the approximate positions by hearing the motor sounds. The author also mentioned that it was more important to know the moving directions of the steps without touching the belts. The author appreciated the proposed system to be able to compensate the hearing sense. These comments indicate that the system is effective in assisting the visually impaired.

In this paper, we performed only the technical experiments in Section 3. Although the blind author appreciated the effectiveness of the proposed system, it is not guaranteed that the proposed system is effective for all visually impaired individuals. However, the experimental results can imply that the system would be able to help many users find available escalators effectively. In this paper, we mainly proposed the system from the viewpoint of system development. In the future, we should evaluate its effectiveness with actual visually impaired individuals, especially blind people.

## 5. Conclusions

This paper proposed a smartphone-based assistive system to enable visually impaired individuals to use escalators safely. The system can detect escalators and determine their movement directions from optical flows in videos obtained by a smartphone camera. The system was evaluated in actual scenes that involved escalators and other objects. The experimental results demonstrate that the system is promising in term of helping visually impaired individuals use escalators.

## Figures and Tables

**Table 1:** Recognition results at 3 m.

		Output				
		$E_{\mathrm{TU}}$	$E_{\mathrm{TL}}$	$E_{\mathrm{FU}}$	$E_{\mathrm{FL}}$	Others
	$E_{TU}$	18	0	0	0	0
	$E_{TL}$	0	14	0	0	4
Input	$E_{FU}$	0	0	17	1	0
	$E_{FL}$	0	0	0	13	5
	Others	0	0	0	0	12

**Table 2:** Recognition results at 5 m.

		Output				
		$E_{\mathrm{TU}}$	$E_{\mathrm{TL}}$	$E_{\mathrm{FU}}$	$E_{\mathrm{FL}}$	Others
	$E_{TU}$	17	1	0	0	0
	$E_{TL}$	0	16	0	0	2
Input	$E_{FU}$	0	0	18	0	0
	$E_{FL}$	0	0	0	16	2
	Others	0	0	0	0	12
